# Establishing the National Institute for Research in Environmental Health, India

**DOI:** 10.2471/BLT.21.286680

**Published:** 2022-02-22

**Authors:** Tanwi Trushna, Rajnarayan R Tiwari

**Affiliations:** a National Institute for Research in Environmental Health, Indian Council of Medical Research, Bhopal Bypass Road, Bhauri-462030, Madhya Pradesh, India.

## Abstract

**Problem:**

Multiple environmental health issues resulting from pollution and climate change threaten public health in India.

**Approach:**

The Government of India recognized the need for a permanent environmental health research institute; the Indian Council of Medical Research therefore established the National Institute for Research in Environmental Health in Bhopal in 2010. Scientists at the institute assessed the multiple long-term health effects of exposure to methyl isocyanate, and are now conducting research on a wide array of locally relevant environmental health issues.

**Local setting:**

The Union Carbide India Limited pesticide factory in Bhopal was the site of a methyl isocyanate gas leak in 1984, which affected half a million people. The Indian Council of Medical Research set up a coordinating unit in the immediate aftermath, which was upgraded to the Bhopal Gas Disaster Research Centre in 1986 and then the Centre for Rehabilitation Studies in 1995.

**Relevant changes:**

Scientists at the institute undertake environmental monitoring and health risk assessment studies among communities located near polluted areas, such as industrial areas. They are also assessing the training needs of practising physicians, with the aim of developing a curated curriculum to meet the deficiencies in environmental health education in the country.

**Lessons learnt:**

Environmental legislation was introduced in the wake of the disaster and a research institute in environmental health was established. Researchers at the institute have recognized the importance of engaging communities in environmental health research, as well as knowledge dissemination to relevant stakeholders.

## Résumé

**Problème:**

La pollution et le changement climatique comportent de nombreux risques pour la santé environnementale, mettant en péril la santé publique en Inde.

**Approche:**

Le gouvernement indien a admis qu'un centre de recherche permanent consacré à la santé environnementale était nécessaire. Le Conseil indien de la recherche médicale a donc créé le National Institute for Research in Environmental Health (institut national de recherche en santé environnementale) à Bhopal en 2010. Les scientifiques de l'institut ont évalué les multiples effets qu'entraîne l'exposition à l'isocyanate de méthyle sur la santé à long terme, et étudient maintenant un large éventail de problèmes de santé environnementale observés à l'échelle locale.

**Environnement local:**

L'usine de pesticides d'Union Carbide India Limited à Bhopal fut le théâtre en 1984 d'une fuite de gaz toxique, l'isocyanate de méthyle, qui a touché un demi-million de personnes. Immédiatement après, le Conseil indien de la recherche médicale a instauré une unité de coordination, devenue par après le Bhopal Gas Disaster Research Centre (centre de recherche sur la catastrophe chimique de Bhopal) en 1986 puis le Centre for Rehabilitation Studies (centre d'étude pour la réhabilitation) en 1995.

**Changements significatifs:**

Les scientifiques de l'institut assurent une surveillance environnementale et mènent des études d'évaluation des risques sanitaires dans les communautés résidant à proximité de zones polluées, par exemple des sites industriels. Ils déterminent en outre les besoins en formation des médecins praticiens, afin de développer un cursus capable de répondre aux lacunes qui subsistent en matière d'éducation à la santé environnementale dans le pays.

**Leçons tirées:**

La législation environnementale a été adoptée à la suite de la catastrophe et a débouché sur la création d'un institut de recherche en santé environnementale. Les chercheurs de l'institut ont reconnu l'importance d'inclure les communautés dans les recherches sur la santé environnementale, mais aussi de transmettre les connaissances aux acteurs concernés.

## Resumen

**Situación:**

Los diversos problemas de salud ambiental derivados de la contaminación y el cambio climático amenazan la salud pública en la India.

**Enfoque:**

El Gobierno de la India reconoció la necesidad de contar con un instituto permanente de investigación en salud ambiental; en consecuencia, el Consejo de Investigación Médica de la India creó el National Institute for Research in Environmental Health (Instituto Nacional de Investigación en Salud Ambiental) en Bhopal en 2010. Los científicos del instituto valoraron los múltiples efectos sanitarios a largo plazo de la exposición al isocianato de metilo, y ahora están investigando una gran variedad de problemas de salud ambiental de importancia local.

**Marco regional:**

La fábrica de plaguicidas Union Carbide India Limited de Bhopal fue el lugar donde se produjo una fuga de gas de isocianato de metilo en 1984, que afectó a medio millón de personas. El Consejo de Investigación Médica de la India creó una unidad de coordinación tras la fuga, que se convirtió en el Bhopal Gas Disaster Research Centre (Centro de Investigación de la Catástrofe por Gas de Bhopal) en 1986 y en el Centre for Rehabilitation Studies (Centro de Estudios para la Rehabilitación) en 1995.

**Cambios importantes:**

Los científicos del instituto realizan estudios de monitoreo ambiental y valoración de riesgos para la salud entre las comunidades ubicadas cerca de las áreas contaminadas, como las zonas industriales. También están valorando las necesidades de formación de los médicos en ejercicio, con el objetivo de elaborar un plan de estudios seleccionado para cubrir las deficiencias de la formación en salud ambiental en el país.

**Lecciones aprendidas:**

La legislación ambiental se estableció como consecuencia de la catástrofe y se creó un instituto de investigación en salud ambiental. Los investigadores del instituto han reconocido la importancia de involucrar a las comunidades en la investigación de la salud ambiental, así como la difusión de conocimientos a las partes interesadas.

## ملخص

**المشكلة:**

هناك العديد من مشاكل الصحة البيئية الناتجة عن التلوث وتغير المناخ، والتي تهدد الصحة العامة في الهند.

**الأسلوب:**

أدركت حكومة الهند الحاجة لوجود معهد دائم لأبحاث الصحة البيئية؛ ولذلك فقد أنشأ المجلس الهندي للأبحاث الطبية المعهد الوطني لأبحاث الصحة البيئية، في بوبال عام 2010. قيّمَ علماء المعهد الآثار الصحية المتعددة طويلة الأجل والناتجة عن التعرض لميثيل الأيزوسيانات، ويقومون الآن بإجراء أبحاث على مجموعة واسعة من القضايا المحلية ذات الصلة بالصحة البيئية.

**المواقع المحلية:**

كان مصنع مبيدات الآفات التابع لشركة Union Carbide India Limited في بوبال، موقعًا لتسرب غاز ميثيل الأيزوسيانات في عام 1984، مما أثر على نصف مليون شخص. قام المجلس الهندي للأبحاث الطبية بإعداد وحدة للتنسيق بعد ذلك مباشرة، والتي تم تطويرها لتكون مركز بوبال لأبحاث كوارث الغاز في عام 1986، ثم إلى مركز دراسات إعادة التأهيل في عام 1995.

**التغيّرات ذات الصلة:**

يقوم العلماء في المعهد بإجراء دراسات للرصد البيئي، وتقييم المخاطر الصحية، بين المجتمعات الواقعة بالقرب من المناطق الملوثة مثل المساحات الصناعية. كما يقومون بتقييم الاحتياجات التدريبية للأطباء الممارسين، بهدف تطوير منهج منظم للتغلب على الثغرات في التعليم الصحي البيئي في الدولة.

**الدروس المستفادة:**

تم إدخال التشريعات البيئية في أعقاب الكارثة وإنشاء معهد لأبحاث الصحة البيئية. لقد أدرك الباحثون في المعهد أهمية إشراك المجتمعات في أبحاث الصحة البيئية، وكذلك نشر المعرفة لأصحاب المصلحة المعنيين.

## 摘要

**问题:**

由污染和气候变化导致的多种环境卫生问题威胁着印度的公众健康。

**方法:**

印度政府意识到有必要设立永久性的环境卫生研究机构；因此，印度医学研究理事会于 2010 年在博帕尔设立了国家卫生研究所。研究所的科学人员评估了异氰酸甲酯暴露所带来的多种长期健康影响，目前正在对当地相关的环境卫生问题开展各项研究。

**当地状况:**

美国联合碳化物公司 (Union Carbide) 公司在印度博帕尔的化工厂于 1984 年发生异氰酸甲酯毒气泄漏事件，影响了 50 万人。印度医学研究理事会在事件发生后立即成立协调小组，该小组于 1986 年被升级为博帕尔毒气灾害研究中心，然后在 1995 年被升级为康复研究中心。

**相关变化:**

研究所的科学人员对污染区（如工厂）附近的社区开展了环境监测和健康风险评估。他们还评估了执业医生的培训需求，目的是精心制定培训课程来改善该国在环境卫生教育方面的不足。

**经验教训:**

灾难发生后出台了环境法规，并设立了环境卫生研究机构。机构中的研究人员意识到参与社区环境卫生研究工作以及向利益相关者宣导教育的重要性。

## Резюме

**Проблема:**

Загрязнение окружающей среды и изменения климата создают многочисленные проблемы в сфере здравоохранения Индии.

**Подход:**

Правительство Индии осознало потребность в создании постоянного института, занимающегося исследованиями в области экологической медицины; в соответствии с этим Индийский совет по медицинским исследованиям принял решение о создании в 2010 году Национального института исследований в области экологической медицины в Бхопале. Ученые этого института занимаются оценкой многочисленных долгосрочных эффектов воздействия метилизоцианата на здоровье и проводят исследования в широком спектре местнозначимых проблем в сфере экологической медицины.

**Местные условия:**

На заводе по производству пестицидов Union Carbide India Limited в Бхопале в 1984 году произошла утечка газообразного метилизоцианата, вследствие чего пострадали около полумиллиона человек. Индийский совет по медицинским исследованиям немедленно после катастрофы создал координационный блок, который со временем был преобразован в Центр исследований газовой катастрофы в Бхопале (1986 г.) и затем в Центр реабилитационных исследований в 1995 г.

**Осуществленные перемены:**

Ученые института предприняли мониторинг окружающей среды и провели исследования с оценкой рисков среди групп населения, проживающих вблизи промышленных зон. Они также оценили потребность в обучении практикующих врачей с целью разработки рекомендованного учебного плана для ликвидации пробелов в сфере образования по вопросам экологической медицины.

**Выводы:**

Законодательство в области окружающей среды, которое было принято после катастрофы, привело к созданию института исследований в области экологической медицины. Исследователи из данного института признали важность привлечения местного населения к исследованиям в области экологической медицины, а также важность распространения знаний среди соответствующих партнеров.

## Introduction

Multiple environmental issues ranging from pollution to climate change threaten public health worldwide. According to the 2016 World Health Organization (WHO) report, an estimated 24% of global mortality and 28% of mortality in children younger than 5 years are attributable to environmental risk factors.^1^ Air pollution is directly responsible for approximately 7 million annual deaths around the globe.^1^ Inadequate access to drinking water and poor sanitation are the causes of an estimated 58% of all cases of diarrhoea in low- and middle-income countries, resulting in 0.8 million premature deaths annually.^1^ WHO has predicted that the effects of climate change will lead to an additional 250 000 deaths every year from 2030 to 2050 as a result of higher numbers of cases of climate-sensitive diseases such as malnutrition, malaria, diarrhoea and heat stress.^2^

The burden of mortality and morbidity attributable to environmental risk factors including climate change is especially significant in low- and middle-income countries,^1^^–^^3^ where 82% of the total global population resides.^3^ In India, many cities have observed high annual concentrations of particulate matter and corresponding high morbidity as a result of air pollution.^4^ According to the 2019 report by the National Institution for Transforming India, many regions in India are currently experiencing water stress (affecting almost 820 million people) and 70% of surface water across the country is polluted, endangering the health and lives of almost 8 million Indian children.^5^

Improvement in public health via improvement in the quality of the environment is one of the most pressing issues for low- and middle-income countries, including India. By means of its sustainable development goals, the United Nations is currently prioritizing global action to meet human developmental needs while minimizing environmental degradation, consequently protecting health.^6^ Accordingly, high-income countries have been actively involved in environmental health research, while many low- and middle-income countries still need to establish environmental health research institutes. The National Institute for Research in Environmental Health, established by the Indian government in 2010, is unique in being one of the few institutes in South-East Asia solely dedicated to environmental health research, a topic that has only recently begun to be addressed within the wider scientific community.^7^ Here we describe setting up the institute and its work.

## Local setting

In 1984, a methyl isocyanate gas leak at the Union Carbide India Limited pesticide factory in Bhopal affected approximately half a million people, either by immediate or subsequent morbidity or mortality.^8^ The leak is considered to be one of the most severe global industrial disasters. The increased environmental health awareness in India resulting from the Bhopal disaster led to changes not just in science (through the establishment of a national environmental health research institute) but also in legislation. Indian parliament enacted the 1986 Environment Protection Act to encompass the previous 1974 Water Act and 1981 Air Act, amended the 1948 Factories Act in 1987, and passed several new acts, such as the 1989 Hazardous Waste (Management and Handling) Rules, the 1996 Chemical Accidents (Emergency Planning, Preparedness and Response) Rules and the 2005 Disaster Management Act.^9^ Impact assessments of the environmental risks posed by any large-scale project before its establishment, as well as periodical follow-up assessments, became mandatory with the Environmental Impact Assessment Notification of 1994 and its subsequent versions.^10^

## Approach

In the immediate aftermath of the Bhopal disaster, the Indian Council of Medical Research set up a coordinating unit. This unit was upgraded to the Bhopal Gas Disaster Research Centre in 1986 and then the Centre for Rehabilitation Studies under the Bhopal Gas Tragedy Relief and Rehabilitation Department of the Government of Madhya Pradesh, in 1995.^8^ Treatment of those affected by the disaster required expertise within many fields, including pulmonology, radiology, neurology, immunology, oncology, pregnancy, childbirth and mental health.^8^ In the decades after the Bhopal gas disaster, the Indian government recognized the need for, and value of, a permanent environmental health research institute. This recognition is reflected in a resolution passed on 24 June 2010 and, on 11 October 2010, the Indian government established the National Institute for Research in Environmental Health in Bhopal under the ambit of the Indian Council of Medical Research.^11^ The institute was set up with a broad mandate of research in environmental health topics affecting the Indian population, including the long-term health effects of the Bhopal gas disaster. 

Initially the institute started functioning with recruitment of nominal staff and the administrative process was initiated to recruit more human resources and to develop the laboratory infrastructure. The State government of Madhya Pradesh provided the initial infrastructure at the old premises at Kamla Nehru Hospital and the land for the construction of a new, bigger campus. In the initial phase most of the projects were related to the health of the gas tragedy survivors, implemented by collaborative efforts of researchers from this institute and other organizations. After obtaining administrative approval, the recruitment procedure was started following the guidelines adopted by the Indian Council of Medical Research. The council provided relevant induction training to newly recruited scientists in matters related to relevant scientific and administrative work, whereas other technical and administrative staff were trained by senior staff working in the institute. As research activities increased with the advent of new scientists, relevant equipment was purchased and laboratories were established through intramural funds provided by the Indian Council of Medical Research as well as by using funds awarded to individual scientists as research grants. 

In the meantime, the permanent campus of the institute was built, with a budget of 1240 million Indian rupees (equivalent to 17.89 million United States dollars) from the Indian government. As a role model to the nation in the adoption of sustainable development practices, the permanent campus of the institute was constructed following the tenets of the Indian Green Building Council to maximize energy and water conservation, while safeguarding the health of the workforce as well as the surrounding environment.^12^ The campus minimizes its negative effects on the environment and ecosystem with efficient water and waste management strategies (e.g. rainwater harvesting, single-use plastic ban), energy efficiency (e.g. motion-sensitive lighting, maximum use of natural light, minimum outdoor light pollution) and green landscaping.^12^ The Union Minister of Health and Family Welfare inaugurated and dedicated the new green campus of the institute to the nation at a ceremony on 13 March 2021.^13^

## Relevant changes

Researchers at the institute recruited and followed up a cohort of individuals affected by the Bhopal disaster to assess the multiple long-term health effects of exposure to methyl isocyanate.^14^ They are now conducting research on a wide array of locally relevant environmental health issues including: (i) molecular-level research to understand the effect of exposure to air pollutants at the epigenetic level; (ii) nanotechnology-based point-of-care diagnostics for pollution-induced diseases such as lung cancer; and (iii) epidemiological studies to understand the burden and distribution of environmental issues such as air pollution, crop residue burning, built environments, noise pollution and chemical water pollution at regional and national levels. 

Since 2016, scientists of the institute have been undertaking environmental monitoring and health risk assessment studies among communities located near industrial areas. They are also assessing the training needs of practising physicians, with the aim of developing a curated curriculum to meet the deficiencies in environmental health education in the country. The need for national research capacity-building in the field of environmental health has been identified; researchers of the new institute therefore provide relevant short-term training programmes both in basic science and public health.

The idea of green social responsibility was promoted at the inauguration ceremony of the institute, based on Principle 10 of the 1992 Rio Declaration on Environment and Development. Such a policy requires public outreach and knowledge dissemination to generate motivation within communities to adopt pro-environmental behaviour. By encouraging communities to make simple changes to their daily activities (e.g. reducing water waste, separating recyclable and compostable waste from landfill, and switching off electrical appliances when not in use), they can reduce their negative impact on the environment. The Indian government is eager to adopt a similar stance to policy-makers in high-income countries that are already developing interventions to encourage behaviour change.^15^ Therefore, scientists of the institute are actively engaging the community in research to educate and empower them to adopt pro-environmental behaviour, while also attempting to educate other Indian public health researchers in the context of community-based participatory research by organizing training courses.

## Lessons learnt

The institute now has an essential role to play in conducting biomedical research on environmental health issues, assessing exposure to environmental pollutants, advocating for relevant mitigation measures and developing the capacity of India in promoting environmental health. The gas tragedy highlighted the disastrous effects of exposure to chemical pollutants on human health. Therefore, the scientists at the institute conduct research among communities residing in potential high pollution zones such as industrial areas (Box 1).

Box 1 **Summary of main lessons learnt**

- Researchers at the new institute now conduct environmental monitoring and health risk assessment studies among communities residing in potential high pollution zones such as industrial areas.
- The institute has identified deficiencies in environmental health education, as well as the need for a national research capacity in the field of environmental health.
- Researchers have recognized the importance of engaging communities in environmental health research, as well as the dissemination of knowledge to relevant stakeholders.

In the initial phase, as the institute lacked sufficient human resources, space and equipment, most of the conducted research was of an epidemiological or survey-based nature and was mostly restricted to the health effects of the gas tragedy. Gradually, with procurement of environmental monitoring equipment, recruitment of specialist scientists and finally the availability of the new, bigger campus with ample space to develop laboratory infrastructure, the institute has started diversifying its research focus into multiple environmental health issues such as air and water pollution, waste management and climate change.

In the future, the institute aims to initiate, through collaboration with other institutes under the Indian Council of Medical Research and reputed medical colleges in the country, a pan-India five-yearly cycle of cross-sectional surveys at province-level to collect information on key health, environment and nutrition-related parameters in the Indian population. Collection of such data will help to fill the gaps in current literature, in addition to assisting with policy-making. Similarly, the scientists of the institute are working to establish urban and rural cohorts to assess the long-term effects of exposure to environmental pollutants specific to the Indian context. 
